# Usability and Acceptability of a Mobile App for the Self-Management of Alcohol Misuse Among Veterans (Step Away): Pilot Cohort Study

**DOI:** 10.2196/25927

**Published:** 2021-04-08

**Authors:** Carol A Malte, Patrick L Dulin, John S Baer, John C Fortney, Anissa N Danner, Aline M K Lott, Eric J Hawkins

**Affiliations:** 1 Center of Excellence in Substance Addiction Treatment and Education (CESATE) Veterans Affairs Puget Sound Health Care System Seattle, WA United States; 2 Health Services Research & Development Seattle Center of Innovation for Veteran-Centered and Value-Driven Care Veterans Affairs Puget Sound Health Care System Seattle, WA United States; 3 Department of Psychology University of Alaska Anchorage Anchorage, AK United States; 4 Department of Psychology University of Washington Seattle, WA United States; 5 Department of Psychiatry and Behavioral Sciences University of Washington Seattle, WA United States

**Keywords:** mobile apps, alcohol misuse, smartphone, veterans, access

## Abstract

**Background:**

Alcohol misuse is common among Operation Enduring Freedom and Operation Iraqi Freedom veterans, yet barriers limit treatment participation. Mobile apps hold promise as means to deliver alcohol interventions to veterans who prefer to remain anonymous, have little time for conventional treatments, or live too far away to attend treatment in person.

**Objective:**

This pilot study evaluated the usability and acceptability of Step Away, a mobile app designed to reduce alcohol-related risks, and explored pre-post changes on alcohol use, psychological distress, and quality of life.

**Methods:**

This single-arm pilot study recruited Operation Enduring Freedom and Operation Iraqi Freedom veterans aged 18 to 55 years who exceeded National Institute on Alcohol Abuse and Alcoholism drinking guidelines and owned an iPhone. Enrolled veterans (N=55) completed baseline and 1-, 3-, and 6-month assessments. The System Usability Scale (scaled 1-100, ≥70 indicating acceptable usability) assessed the effectiveness, efficiency, and satisfaction dimensions of usability, while a single item (scaled 1-9) measured the attractiveness of 10 screenshots. Learnability was assessed by app use during week 1. App engagement (proportion of participants using Step Away, episodes of use, and minutes per episode per week) over 6 months measured acceptability. Secondary outcomes included pre-post change on heavy drinking days (men: ≥5 drinks per day; women: ≥4 drinks per day) and Short Inventory of Problems–Revised, Kessler-10, and brief World Health Organization Quality of Life Questionnaire scores.

**Results:**

Among the 55 veterans enrolled in the study, the mean age was 37.4 (SD 7.6), 16% (9/55) were women, 82% (45/55) were White, and 82% (45/55) had an alcohol use disorder. Step Away was used by 96% (53/55) of participants in week 1, 55% (30/55) in week 4, and 36% (20/55) in week 24. Step Away use averaged 55.1 minutes (SD 57.6) in week 1 and <15 minutes per week in weeks 2 through 24. Mean System Usability Scale scores were 69.3 (SD 19.7) and 71.9 (SD 15.8) at 1 and 3 months, respectively. Median attractiveness scores ranged from 5 to 8, with lower ratings for text-laden screens. Heavy drinking days decreased from 29.4% (95% CI 23.4%-35.4%) at baseline to 16.2% (95% CI 9.9%-22.4%) at 6 months (*P<.*001). Likewise, over 6 months, Short Inventory of Problems–Revised scores decreased from 6.3 (95% CI 5.1-7.5) to 3.6 (95% CI 2.4-4.9) (*P<.*001) and Kessler-10 scores decreased from 18.8 (95% CI 17.4-20.1) to 17.3 (95% CI 15.8-18.7) (*P*=.046). Changes were not detected on quality of life scores.

**Conclusions:**

Operation Enduring Freedom and Operation Iraqi Freedom veterans found the usability of Step Away to be acceptable and engaged in the app over the 6-month study. Reductions were seen in heavy drinking days, alcohol-related problems, and Kessler-10 scores. A larger randomized trial is warranted to confirm our findings.

## Introduction

Alcohol misuse, which is associated with a number of adverse social, economic, and health-related consequences [1,2], is one of the most common conditions among Operation Enduring Freedom (OEF) and Operation Iraqi Freedom (OIF) service members and veterans, with estimates ranging from 22% to 40% [3-5]. Previous reports suggest the rate of alcohol misuse among OEF and OIF veterans is 2 times the rate observed for similarly aged veterans who did not serve in OEF and OIF and much higher among men (22%) than women (5%) [5]. Despite high rates of use, many OEF and OIF veterans with alcohol-related problems do not receive alcohol-related care or only receive care after significant delay [6-8]. The large gap between those who need and those who receive treatment is thought to be due in part to barriers to using available services [9].

OEF and OIF veterans have reported several impediments to seeking mental health care, including stigma-related barriers, such as beliefs that they will be perceived as weak for seeking help, and logistical barriers, such as insufficient time, burdensome paperwork, and long distances to the nearest treatment facility [3,10,11]. Mobile apps, delivered on smartphones, have the potential to address the majority of these barriers, as they can deliver alcohol interventions to those who are interested in help but prefer to remain anonymous, have little time for traditional therapy, or must travel too far to attend treatment in person [12]. Unlike traditional alcohol interventions that rely on patient-to-provider encounters, mobile apps deliver timely interventions to individuals in their natural settings at crucial moments when the need for intervention is high and repeatedly over time [13]. Further, smartphone ownership in the United States is common, particularly among persons aged 18 to 49 years [14], and there is considerable interest in tools that allow for self-management of alcohol use [15].

While mobile apps to manage alcohol use have proliferated in recent years, they vary greatly in quality, and many are narrow in scope (ie, limited to tracking of alcohol consumed, estimating blood alcohol content [15]) or lack key evidence-based intervention components associated with behavior change (ie, normative feedback, use of social support [16]). While data on the effect of apps, particularly those aimed at young adults, on drinking outcomes are growing [17,18], limited information exists on their acceptability and usability, particularly among OEF and OIF veterans [19,20]. The importance of collecting this type of information is highlighted by a recent qualitative study of rural veterans [21], which found that these veterans held more negative views of apps relative to urban veterans and expressed that apps were hard to navigate, hard to access due to connectivity issues, and opposed to their values (impersonal, lacking connection to community). App acceptability and usability are closely related to engagement, a key issue in app development, with several studies finding that app participation drops off steeply after 1 week, with only the most at-risk and motivated individuals remaining engaged (eg, Drinkaware [22]). The relationship between app engagement and drinking outcomes is not well established. A small study of an app (InDEx) developed for British ex-service members showed promise in engaging participants over the 4-week study course, with modest reductions in drinking [23]. A second study found that age, gender, and education rather than baseline drinking levels were associated with app (Drink Less) engagement in a nonveteran sample; however, engagement with the app was not associated with drinking outcomes [24].

Two of the most studied and comprehensive smartphone-based interventions are the Location-Based Monitoring and Intervention System for Alcohol Use Disorders (LBMI-A) and the Addiction Comprehensive Health Enhancement Support System (A-CHESS). Designed to provide stand-alone treatment, LBMI-A has been evaluated among a small community sample who met Diagnostic and Statistical Manual of Mental Disorders, Fifth Revision (DSM-5) criteria for an alcohol use disorder (AUD). Results suggest a significant reduction in the percentage of heavy drinking days (HDD) from 56% at baseline to 25% at 6 weeks [25,26]. A substantial barrier to engagement with LBMI-A was the reported difficulty in simultaneously using a personal and LBMI-A–enabled phone in the pilot study (prior to mobile apps’ emergence). In contrast to LBMI-A, A-CHESS was designed to prevent relapse to heavy drinking among patients who completed residential treatment for an AUD [27]. A-CHESS patient-generated data are accessed by treatment providers to monitor patent progress; as such, it is not anonymous or independent of traditional treatment.

Step Away, the next iteration of LBMI-A, is an iOS-based mobile app designed to help people self-manage drinking and alcohol-related problems and thus is an alternative for those who do not want or have access to traditional treatment [12]. A recent systematic review of eHealth interventions for alcohol identified several recommendations [20], such as addressing the spectrum of alcohol use (at-risk drinking to AUD), including cognitive behavioral coping strategies and access to timely interventions for skill development and navigation of high-risk situations, and using mobile technologies such as push notifications to promote engagement. Step Away complies with many of these recommendations by addressing a range of severity of alcohol-related problems; providing education, advice, and goal setting related to alcohol use; soliciting participant data on drinking, craving, and mood through push notifications; and compiling these data in regular feedback reports.

This mixed methods pilot study was designed to evaluate the usability and acceptability of Step Away among OEF and OIF veterans with alcohol misuse who are not involved in traditional alcohol treatment. The primary aim of this project was to assess the usability of Step Away among OEF and OIF veterans. According to the International Organization for Standardization Guidance on Usability [28], key dimensions of usability are efficiency, effectiveness, and satisfaction. Further, a recent review of mobile health (mHealth) apps [29] recommends the assessment of attractiveness and learnability, important usability dimensions that are infrequently evaluated in the literature. A second aim was to assess the acceptability of the app, which was measured by engagement. Exploratory aims were to evaluate change in alcohol use, psychological distress, and health-related quality of life outcomes associated with the use of Step Away over the 6-month study course and to examine associations between app engagement, baseline characteristics, and drinking outcomes.

## Methods

This single-arm prospective cohort study involving a national sample of OEF and OIF veterans (n=55) occurred between September 2017 and December 2018. The study was approved by the Veteran Affairs (VA) Puget Sound Institutional Review Board.

### Study Participants

OEF and OIF veterans were eligible to participate if they (1) were aged 18 to 55 years, (2) screened positive for alcohol misuse (≥5 standard drinks on any day or ≥15 standard drinks per week for men and ≥4 standard drinks on any day or ≥8 standard drinks per week for women) in the prior 4 weeks, and (3) owned an iPhone. Exclusion criteria included (1) participation in substance use treatment, including Alcoholics Anonymous, in the past 30 days; (2) pregnancy; and (3) satisfaction of DSM-5 criteria for a drug use disorder (nicotine use disorders and drug disorders in remission allowed) or psychotic disorder.

### Procedures

The planned strategy of recruiting participants using social media advertisements proved unsuccessful, with advertisements yielding no contacts from interested participants after 3 months. Subsequently, investigators used the VA Corporate Data Warehouse, a data repository of patient-level data from the VA electronic medical record, to identify OEF and OIF veterans with Alcohol Use Disorders Identification Test–Consumption [30] scores of 5 or greater (indicating alcohol misuse) who used VA care in the past 6 months (n=13,266) for recruitment by letters and follow-up contacts. Potentially eligible veterans were randomly selected and, in blocks of 100, mailed recruitment letters introducing the study as an evaluation of an app designed to improve the health of individuals who drink alcohol. A total of 10% of letters were sent to women to ensure adequate sampling. Research staff then attempted to contact veterans weekly for up to 3 weeks via telephone and conducted brief telephone screens to assess eligibility with those who were reached and interested in participation. Eligible and interested veterans were mailed a consent form and completed informed consent procedures by telephone. Following consent, veterans completed a baseline research assessment and then received instructions on how to download Step Away to their smartphone. Follow-up assessments occurred at 1, 3, and 6 months. A subset of veterans completed qualitative interviews during the initial 3 months (data to be reported separately). All assessments were completed by telephone, with secure messaging (SMS) used to schedule and remind participants about appointments. Participants were compensated US $40 for completing baseline and 3- and 6-month follow-up assessments and US $5 for completing a usability questionnaire at the 1- and 3-month follow-up.

### Intervention: Step Away Mobile App Content and Features

A version of the Step Away app similar to the one used by this study has been described in detail elsewhere [31]. Briefly, Step Away is a comprehensive intervention for alcohol misuse based on the evidence-based principles of motivational enhancement therapy (MET) [32], relapse prevention [33], and community reinforcement [34,35]. Consistent with a harm reduction approach that supports motivation to change in a nonjudgmental, facilitative manner, Step Away supports both reductions in and abstinence from drinking. Step Away incorporates relapse prevention strategies to identify and cope with situations that increase the risk of relapsing or drinking inconsistently with goals as well as community reinforcement strategies that encourage involvement of supportive others in setting drinking goals and participating in nondrinking activities.

In total, Step Away consists of 10 modules: drinking profile, goal setting, rewards, cravings, strategies, supportive others, reminders, high-risk times, moods, and new activities. Taken together, these modules encompass assessment, normative feedback, goal setting, behavioral strategies tailored to the goals of moderation or abstinence, involvement of supportive others, and replacement activities. Users can personalize the app through the use of reminders about reasons for changes, high-risk times, and scheduled activities. It is anticipated that app users will spend more time in the app during the initial session to review content and set up goals and personal preferences.

Using push notifications, Step Away prompts users daily to complete a brief self-monitoring questionnaire on current mood, drinking behaviors, and alcohol cravings and triggers for alcohol use during the prior 24 hours. Step Away provides weekly feedback, highlighting progress toward goals, ongoing cravings and triggers, and moods experienced throughout the prior week. Feedback includes strategies for managing common alcohol cravings and triggers and a feature for users to enter future high-risk events that might negatively influence drinking goals. Step Away provides real-time intervention options, including strategies for managing cravings or negative emotions, requests for help (eg, to manage anxiety or alcohol cravings), and working through a problem via a problem-solving algorithm [36]. Veterans are provided with an option to contact the Veterans Crisis Line if in crisis and VA or non-VA addiction programs if more traditional treatment services are desired.

### Measures and Outcomes

Study data included both patient self-report and data generated automatically through the use of Step Away. Assessments are detailed below. Step Away use data were automatically generated and stored on secure Step Away servers. Data included date and time of session start and end, specific screen views, time spent per screen view, use of Step Away features, and completion of daily brief assessments.

#### Eligibility and Patient Characteristics

The brief telephone screen assessed age, iPhone ownership, OEF or OIF service, pregnancy, current participation in VA or non-VA addiction treatment, recent alcohol and drug use, and potential psychotic symptoms. Positive screens for drug use or psychotic symptoms were followed up with the appropriate modules of the Mini International Neuropsychiatric Interview (MINI) modified for the DSM-5 [37]. At baseline, demographics and the MINI depressive, bipolar, anxiety, alcohol use, and posttraumatic stress disorders modules were completed.

#### Primary Outcomes

The main study outcome was app usability, comprised of effectiveness, efficiency, satisfaction, learnability, and attractiveness. Effectiveness measures the ability of users to perform a given task, efficiency describes the resources (eg, time) expended to perform the task after it has been learned, and satisfaction represents users’ assessments of how well the device met their needs. These 3 dimensions of usability were assessed at the 1- and 3-month follow-up using the System Usability Scale (SUS; scaled 1 to 100), a 10-item, well-validated questionnaire [38] with scores of ≥70 considered acceptable [39]. Learnability represents users’ abilities to accomplish a task on their first attempt, which is an important determinant of engagement. The learnability of Step Away was measured by the total time spent in the app on the first episode of use and the first week of use. Further, an analysis of the SUS validated a 2-factor structure capturing learnability and usability [40]. Similar to the full SUS, both components are scaled from 0 to 100. Additionally, the attractiveness of Step Away was assessed at 1 month using a validated 9-point Likert scale item (scaled 1=low to 9=high) designed to assess the visual appeal of web pages [41]. Median scores on the 1-item questionnaire measured attractiveness of 10 distinct Step Away screenshots.

Acceptability was measured through app engagement [42] and included the following measures derived from Step Away use data: proportion of participants who used Step Away, number of episodes of use, and episode length (time between opening and closing of app) overall and by week during the 6 months after baseline assessment. We also calculated the number of times each module was viewed and the number of times daily questionnaires were completed by each participant. Means (standard deviations) and medians (interquartile ranges) were used to calculate these measures.

#### Secondary Outcomes

Secondary outcomes included change in alcohol use, psychological distress, and health-related quality of life from baseline to month 6. Assessments collected at baseline and 3- and 6-month follow-up included (1) the Timeline Followback (TLFB) [43] to assess alcohol use in the prior 30 days and number of standard drinks (14 grams of alcohol) per day; (2) the Short Inventory of Problems–Revised (SIP-R) [44] to assess drinking-related consequences; (3) the Kessler-10, a reliable and valid 10-item psychological distress measure (with scores ranging from 10 to 50), to asses distress over the prior month [45,46]; and (4) the abbreviated World Health Organization Quality of Life (WHOQOL-BREF), a 26-item measure derived from the WHOQOL-100 quality of life measure, to assess 4 domains related to quality of life, that is, physical health, psychological health, social relationships, and environment [47].

Alcohol use outcomes were defined as changes in the percentage of HDD (≥5 drinks per day for men; ≥4 drinks per day for women) in the prior 30 days and the proportion of patients drinking below the recommended limits, defined as <15 drinks per week and <5 drinks per day for men and <8 drinks per week and <4 drinks per day for women, as determined by TLFB data. Change in heavy drinking days was selected because it is a marker of alcohol misuse, associated with long-term health outcomes, and frequently used as an outcome in the alcohol treatment literature [48,49]. Alcohol consequences were measured by change in the SIP-R total score. Changes in the Kessler-10 total score and 4 domain scores of the WHOQOL-BREF measured psychological distress and health-related quality of life outcomes, respectively.

### Analyses

Usability, learnability, attractiveness, and engagement outcomes as well as participant characteristics are presented using descriptive statistics, including means with standard deviations and medians with interquartile ranges. Associations between app engagement (total weeks and minutes of use) and demographics, baseline alcohol use, drug use, and mental health diagnoses were examined using unadjusted linear regression. Responses at baseline and 3 and 6 months represent repeated measures of clinical outcomes over time. Longitudinal analyses using multilevel mixed models were used to estimate the percent change in HDD between baseline and month 6, with a fixed effect for time and a unique patient identifier included as a random effect. Gender, age, and binary measures of AUD, any drug use, and mental health disorders as per the MINI (major depression, generalized anxiety, and posttraumatic stress disorder) at baseline were included as covariates to determine their associations with changes in the percentage of HDD. A similar approach was used to estimate changes in alcohol consequences (SIP-R), distress (Kessler-10), and quality of life (WHOQOL-BREF) outcomes. The proportion of participants drinking below guideline limits at follow-up was examined using a Pearson chi-square test. Associations between change over time in alcohol-related outcomes (HDD, SIP-R) and app engagement were explored using multilevel mixed models that included time, a measure of app engagement (total weeks or minutes of use), and the interaction between time and app engagement, with a patient identifier included as a random effect. All analyses were conducted with Stata MP (version 15; StataCorp).

## Results

### Participants

**Figure 1:**
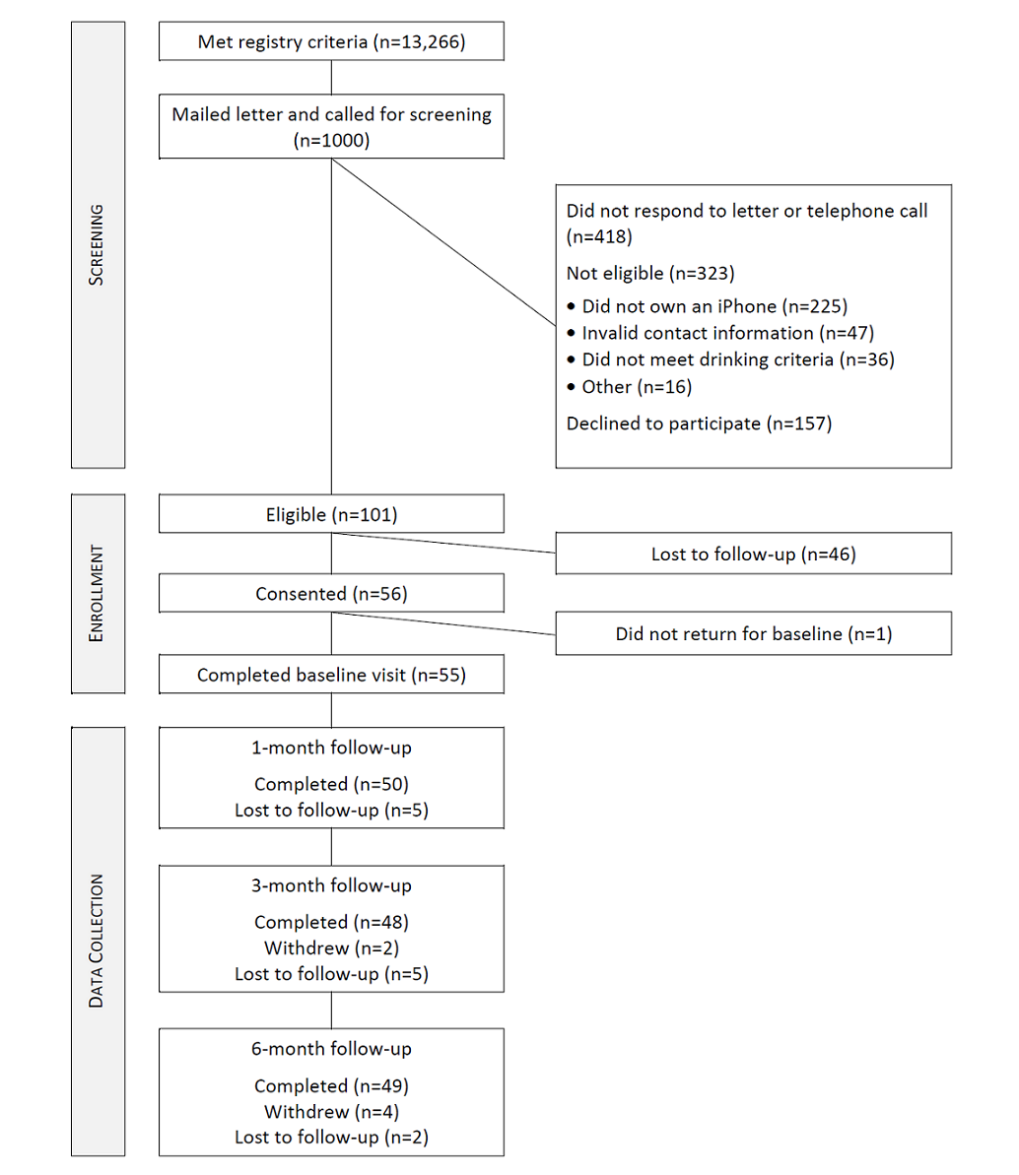
CONSORT diagram.

As shown in Figure 1, over 140 days, 1000 potentially eligible veterans were invited to participate by mail. Of this group, 621 (62.1%) veterans did not respond or were unable to be contacted by telephone (418/1000, 41.8%) or declined to participate (157/1000, 15.7%), and 324 (32.4%) screened ineligible (eg, did not own an iPhone). Of the remaining 101 veterans, 55 enrolled in the study. Completion rates of follow-up assessments were over 85% at all time points. Participants’ mean age was 37.4 (SD 7.6), 16% (9/55) were women, and 82% (45/55) were White (Table 1). A total of 82% (45/55) of participants met DSM-5 criteria for an alcohol use disorder and 33% (18/55) had received treatment for a substance use disorder in the past.

**Table 1:** Participant characteristics (N=55).

Characteristic			Value
Age, mean (SD)			37.4 (7.6)
Female, n (%)			9 (16.4)
**Race, n (%)**			
	White	45 (81.8)	
	Black	4 (7.3)	
	Asian or Pacific Islander	3 (5.5)	
	American Indian	3 (5.5)	
	Hispanic^a^	10 (18.5)	
**Education (years), mean (SD)**			
	12	11 (20.0)	
	13-15	18 (32.7)	
	16+	26 (47.3)	
**Marital status, n (%)**			
	Married or living with partner	36 (65.4)	
	Divorced or separated	8 (14.6)	
	Never married	11 (20.0)	
Currently employed, n (%)			43 (78.2)
**Income (US $), n (%)**			
	0 to 10,000	2 (3.6)	
	10,000 to 50,000	25 (45.5)	
	50,000 to 100,000	21 (38.2)	
	>100,000	7 (12.7)	
Prior substance use disorder treatment, n (%)			18 (32.7)
Any drug use, n (%)			2 (3.6)
**Mental health conditions, n (%)**			
	Current major depressive disorder	4 (7.3)	
	Generalized anxiety disorder	3 (5.5)	
	Posttraumatic stress disorder	5 (9.1)	
	Alcohol use disorder	45 (81.8)	

^a^N=54.

### Usability of Step Away

Participants’ mean SUS scores, which assess effectiveness, efficiency, and satisfaction, were 69.3 (SD 19.7) and 71.9 (SD 15.8) at the 1- and 3-month follow-up, respectively, with 62% (31/50) and 77% (36/47) of participants scoring ≥70 points at the 2 time points. Learnability was assessed by time taken to complete modules the first time. Participants spent an average 19.2 (SD 25.1) minutes in Step Away for the first episode of use compared to an average of 11.9 (SD 10.6) minutes per episode during all of week 1 and less than a minute per episode at week 24. Mean scores on the learnability component of the SUS were 80.0 (SD 22.9) at 1 month and 79.0 (SD 17.3) at 3 months. Of the 10 screenshots that participants evaluated, attractiveness scores ranged from a median of 5 to 8 (out of 9), with lower ratings given to text-laden screens.

### Acceptability of Step Away

**Figure 2:**
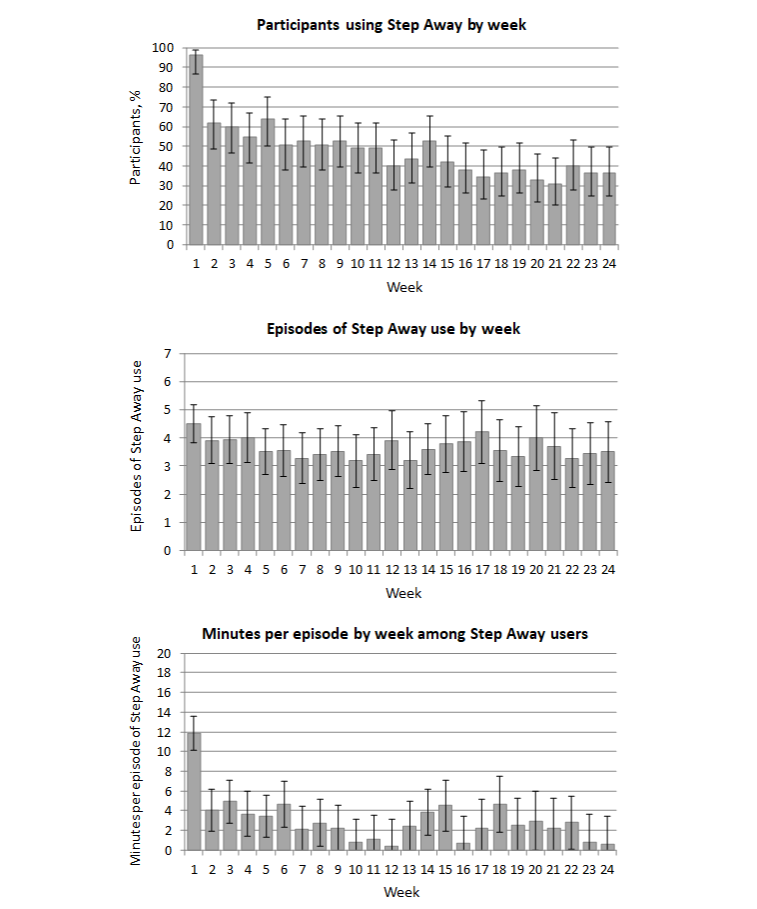
Step Away app engagement by week. App users are defined as study participants who used the app in a given week.

As seen in Figure 2, nearly all participants accessed Step Away in week 1, with the percentage dropping to 62% (34/55) in week 2 and 36% (20/55) by week 24. All 55 participants accessed the app at least once over the 6-month course of the study. A total of 24 of the 55 (44%) participants accessed the app every week during weeks 1 through 4, and 12 (22%) accessed the app every week during weeks 1 through 12. Time spent in the app dropped off considerably after week 1, with participants spending close to 60 minutes (mean 55.1, SD 57.6) in Step Away in week 1 and less than 15 minutes in weeks 2 to 24. However, episodes of Step Away use per week remained at 3 to 4 over the course of the study among participants accessing the app at least once in the given week. The majority of app modules were accessed by ≥60% of participants, and consumption- and tracking-focused modules such as daily feedback were accessed most frequently (median times accessed: 12, IQR 2-52) and by the largest proportion of participants (>80%) (Table 2).

**Table 2:** Participants’ use of Step Away by module from baseline to month 6.

Feature and activity			Ever accessed, n (%)		Number of times accessed		
					Mean (SD)		Median (IQR)
**General overview**							
	Overview and setup	55 (100)		2.3 (1.5)		2 (1-3)	
**Drinking profile module**							
	Initial drinking profile	53 (96.4)		2.9 (2.0)		2 (2-4)	
	Maximum drinks	53 (96.4)		1.6 (1.2)		1 (1-2)	
	Peak BAC^a^	53 (96.4)		1.4 (0.7)		1 (1-2)	
	Dependency and SADQ^b^	49 (89.1)		0.9 (0.4)		1 (1-1)	
	Money and total cost	47 (85.5)		0.9 (0.4)		1 (1-1)	
**Goals module**							
	Goals step	43 (78.2)		2.2 (2.2)		2 (1-3)	
	Goals path (abstinence and moderation)	42 (76.4)		0.9 (0.7)		1 (1-1)	
**Rewards module**							
	Rewards	41 (74.5)		1.3 (1.7)		1 (0-2)	
**Craving module**							
	Craving	35 (63.6)		1.0 (1.0)		1 (0-2)	
**Supportive others module**							
	Supportive person	34 (61.8)		1.4 (1.7)		1 (0-2)	
**Strategies module**							
	Strategies	33 (60.0)		1.1 (1.3)		1 (0-2)	
**Reminders module**							
	Reminder	33 (60.0)		0.8 (0.9)		1 (0-1)	
**Moods module**							
	Mood	34 (61.8)		1.0 (1.3)		1 (0-1)	
	Depression level	33 (60.0)		0.6 (0.6)		1 (0-1)	
	Stress level	33 (60.0)		0.6 (0.6)		1 (0-1)	
**High-risk times module**							
	High-risk accessed	33 (60.0)		0.9 (1.1)		1 (0-1)	
**New activities module**							
	Activities step accessed	33 (60.0)		1.0 (1.1)		1 (0-2)	
	Activities entered	32 (58.2)		0.8 (0.8)		1 (0-1)	
**Ongoing assessment and feedback**							
	Daily feedback finished	45 (81.8)		36.9 (51.7)		12 (2-52)	
	Weekly feedback finished	35 (63.6)		7.3 (9.1)		3 (0-14)	
	Weekly feedback drink total	34 (61.8)		5.8 (7.8)		2 (0-11)	
**In-the-moment tools**							
	Get help accessed	46 (83.6)		2.6 (2.5)		2 (1-3)	
	Help type selected (anxious, craving, down, problem, other call)	15 (27.3)		0.4 (0.8)		0 (0-1)	

^a^BAC: blood alcohol concentration.

^b^Severity of Alcohol Dependence Questionnaire.

Associations with app engagement, as measured by total weeks and minutes of app use over 24 weeks, are shown in Table 3. Total weeks of use was positively associated with female gender, whereas total minutes of app use was positively associated with age, years of education, and income. Associations were not detected between engagement and baseline alcohol use severity, drug use, or mental health comorbidity.

**Table 3:** Associations between participant characteristics and Step Away engagement.

Characteristic		Weeks of use		Total minutes of use	
		β (95% CI)	*P* value	β (95% CI)	*P* value
Age		0.16 (–0.12 to 0.45)	.25	6.46 (1.44 to 11.47)	.01
Female		7.58 (2.42 to 12.74)	.005	50.79 (–63.66 to 165.24)	.38
Person of color		1.00 (–3.72 to 5.73)	.67	30.54 (–56.47 to 117.55)	.49
**Education**					
	12 years	Ref^a^	N/A^b^	Ref	N/A
	13-15 years	3.94 (–1.23 to 9.11)	.13	56.02 (–14.31 to 126.35)	.12
	16+ years	5.12 (–0.08 to 10.32)	.05	109.89 (40.28 to 179.49)	.003
Income >$50,000 (US)		1.00 (–3.55 to 5.55)	.66	88.20 (14.93 to 161.48)	.02
**Baseline characteristics**					
	Count AUD^c^ criteria	–0.61 (–1.33 to 0.10)	.09	–12.07 (–25.74 to 1.59)	.08
	Percent heavy drinking days	–0.01 (–0.08 to 0.06)	.76	–0.26 (–1.41 to 0.89)	.65
	Average drinks per day	–0.35 (–1.18 to 0.48)	.41	–3.39 (–16.08 to 9.30)	.59
	SIP-R^d^ score	–0.10 (–0.49 to 0.29)	.62	–2.33 (–8.42 to 3.76)	.45
	Drug use	–2.01 (–13.09 to 9.07)	.72	–71.88 (–152.04 to 8.28)	.08
	Mental health condition	–0.04 (–6.03 to 5.94)	.99	57.50 (–66.80 to 181.81)	.36

^a^Ref: reference category.

^b^N/A: not applicable.

^c^AUD: alcohol use disorder.

^d^SIP-R: Short Inventory of Problems–Revised.

### Alcohol Use, Psychological Distress, and Health-Related Quality of Life

**Figure 3:**
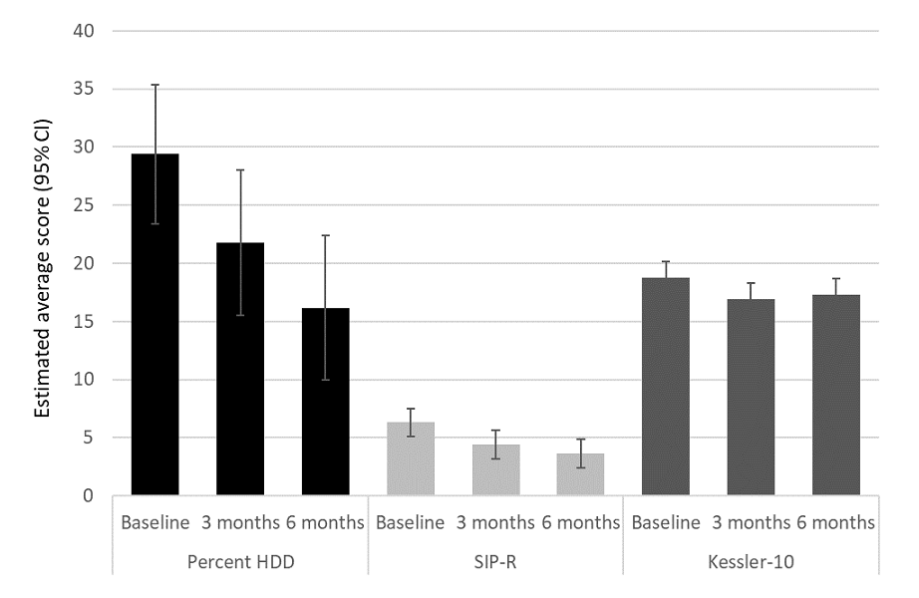
Estimated alcohol use and psychological distress scores over time, adjusted for gender, age, and binary measures of alcohol use disorder, any drug use, and mental health disorder (major depression, generalized anxiety, and posttraumatic stress disorder). HDD: heavy drinking days; SIP-R: Short Inventory of Problems–Revised.

The percentage of participants drinking above recommended limits dropped from 100% (55/55) at baseline to 88% (42/48) at 3 months and 80% (39/49) at 6 months (*P*=.003). Participants’ scores over time are presented in Table 4 and visually displayed in Figure 3. Following adjustment for covariates, decreases were seen in participants’ percentage of HDD in the past 30 days from 29.4% at baseline to 21.8% at 3 months (β=–7.6, 95% CI –13.6 to –1.6; *P*=.01) and 16.2% at 6 months (β=–13.2, 95% CI –19.2 to –7.2; *P<.*001). Likewise, drinking consequences as per the SIP-R decreased from 6.3 at baseline to 4.4 at 3 months (β=–1.9, 95% CI –2.8 to –0.9; *P<.*001) and 3.6 at 6 months (β=–2.7, 95% CI –3.6 to –1.7; *P<.*001). Psychological distress as per the Kessler-10 scores decreased from baseline an estimated –1.9 (95% CI –3.3 to –0.4; *P*=.01) points at 3 months and –1.5 (95% CI –2.9 to 0.0; *P*=.046) points at 6 months. Changes were not detected in any of the WHOQOL-BREF domain scores over the study course. No associations were detected between changes in alcohol-related outcomes and app engagement (total weeks and minutes of app use over 24 weeks).

**Table 4:** Estimated alcohol use, psychological distress, and health-related quality of life scores over time.^a^

Measure			Estimated mean (95% CI)
**Heavy drinking days, %**			
	Baseline	29.4 (23.4-35.4)	
	Month 3	21.8 (15.5-28.0)	
	Month 6	16.2 (9.9-22.4)	
**SIP-R^b^ score**			
	Baseline	6.3 (5.1-7.5)	
	Month 3	4.4 (3.2-5.7)	
	Month 6	3.6 (2.4-4.9)	
**Kessler-10**			
	Baseline	18.8 (17.4-20.1)	
	Month 3	16.9 (15.4-18.3)	
	Month 6	17.3 (15.8-18.7)	
**WHOQOL-BREF^c^, physical health**			
	Baseline	59.5 (57.1-61.9)	
	Month 3	59.1 (56.5-61.6)	
	Month 6	60.2 (57.7-62.8)	
**WHOQOL-BREF, mental health**			
	Baseline	67.9 (64.8-71.0)	
	Month 3	67.5 (64.3-70.7)	
	Month 6	68.0 (64.8-71.2)	
**WHOQOL-BREF, social relationships**			
	Baseline	72.6 (67.4-77.8)	
	Month 3	75.4 (70.0-80.8)	
	Month 6	73.7 (68.3-79.0)	
**WHOQOL-BREF, environment**			
	Baseline	79.9 (76.7-83.1)	
	Month 3	78.2 (74.9-81.5)	
	Month 6	80.1 (76.7-83.4)	

^a^Adjusted for gender, age, and binary measures of alcohol use disorder, any drug use, and mental health disorders (major depression, generalized anxiety, and posttraumatic stress disorder) at baseline.

^b^SIP-R: Short Inventory of Problems–Revised.

^c^WHOQOL-BREF: abbreviated World Health Organization Quality of Life.

## Discussion

### Principal Findings

The low rates of and delays in accessing treatment for alcohol-related problems among OEF and OIF veterans can detrimentally affect their health, long-term functioning, and reintegration into communities. While specialty substance use disorder care is seen as the gold standard for alcohol treatment, mHealth apps such as Step Away have great public health reach [15] and are a promising alternative for veterans who are unable or reluctant to seek traditional clinic-based alcohol treatment. This study contributes to the emerging literature on alcohol treatment apps and to the investigations of Step Away, the next iteration of LBMI-A, in particular. Results from this pilot study indicated that OEF and OIF veterans were willing to engage with Step Away; found the app acceptable with respect to effectiveness, efficiency, and overall satisfaction; and decreased their heavy drinking days over 6 months of study participation.

While veterans recruited for this study were selected based on reports of drinking over recommended limits, they were not required to have an AUD, be seeking care, or have a goal of changing their drinking behavior. Despite these facts, over 80% (45/55) of the sample met criteria for AUD and over one-third had received prior substance use treatment at baseline. Given our sample and the mixed findings from other studies of alcohol-related apps, many showing modest reductions in use [22,23], the decreases seen in heavy drinking days and alcohol-related problems scores are encouraging. Future studies with a larger sample size and a control group are needed to validate these findings and determine their association with Step Away.

Weekly episodes of Step Away use among those who accessed the app at least once during a given week remained steady over the study course, but time per episode dropped quickly after week 1. Such findings are similar or better than those of other studies (eg, 5% of participants using the app at week 12 [22]). This drop-off in use is understandable; we anticipated that most participants would complete the most time-consuming aspects of Step Away (eg, setting up their preferences and goals and reading through psychoeducational material) during week 1. Sustained use consisted mainly of the daily and weekly feedback features of Step Away, which continued to be used by approximately one-third of participants through week 24. This may be driven by Step Away’s use of push notifications, as recommended in the literature [15,20,50]. While these features are important, other components based in MET (eg, goal setting) and community reinforcement (eg, social supports) were not as well accessed.

This raises the question of how to deliver treatment components that are thought to be essential to quality substance use disorder care in the virtual environment. Some apps (eg, A-CHESS) are designed to be adjunctive to standard in-person care, either to be used as an additional component of care or a continuation of care. For stand-alone treatment apps such a Step Away, the issue of how to engage app users with evidence-based interventions is critical. Future iterations of Step Away must leverage technology to deliver these interventions in a way that is perceived as relevant and useful. Personalizing app content (ie, tailoring) is associated with increases in user-rated quality and popularity [15]. Apps such as InDEx [23], which is geared specifically to a veteran population, included highly tailored messaging and saw high rates of app engagement, with two-thirds of participants using the app every week for 4 weeks after enrollment compared to 43.6% (24/55) seen in this study. More sophisticated use of tailoring that introduces new interventions specific to the app user’s treatment course (eg, relapse prevention) might help to keep users fully engaged in app content and reengage those individuals whose app usage has dropped off. Combining Step Away with provider contact, peer support, or both, a recommended means of increasing engagement [51], is under study by Blonigen and colleagues [52] and will be critical to our understanding of how to best improve engagement with alcohol treatment apps.

Similar to findings pertaining to engagement with the DrinkLess app [24], which was tested in the general population, engagement with Step Away as measured by weeks of use and total minutes of use was positively associated with age, female gender, education level, and an income above US $50,000. Examinations of the relationship between drinking characteristics and app engagement have yielded inconsistent results, with some studies identifying an association between high drinking levels and app engagement [22] and others detecting no association [24]. Our findings of no significant associations between baseline drinking severity and engagement must be considered in relation to our sample. The study was presented as an app evaluation rather than a treatment study, and as noted above, participants were not required to have a desire to cut back on alcohol use. It is also possible that engagement reflected a desire to be compliant with the study rather than treatment engagement per se. Our findings also do not help clarify whether app engagement improves drinking outcomes. While we did not detect an association between engagement and outcomes, our sample size was small and likely underpowered. Additional research, ideally with a comparison condition and a larger sample of participants desiring to cut back on use, is needed to determine whether Step Away is more appealing to veterans with alcohol misuse and no AUD compared with those with AUD and to assess the relationship between app engagement and outcomes.

### Study Limitations

While the primary aim of this study was to evaluate the usability of Step Away, we were not able to tease out the main components of usability—effectiveness, efficiency, and satisfaction—because the SUS is a composite measure. In addition, we were unable to determine with accuracy the amount of time spent in each Step Away module for the first time. Thus, learnability was assessed by time spent in the app on the first episode of use and first week of use, with time spent in the app after week 1 provided for context. The study relies on veteran self-report for measuring alcohol use and prior receipt of alcohol-related care without verification by laboratory testing and treatment utilization records. Without a comparison condition, we do not know if reductions in use were due to use of Step Away or other factors; other studies of alcohol treatment apps [17] have found that reductions in use are modest when compared to controls and that differences decline over time. Furthermore, we did not assess participants’ motivations for using the app or whether they desired or saw a need for cutting back on alcohol consumption. While we assessed general distress using the Kessler-10, we did not examine symptoms related to co-occurring mental health conditions that are common among veterans (eg, depression, posttraumatic stress disorder). As iPhone users tend to be less racially diverse and report higher incomes than other smartphone users, the sample of participants recruited for this study is not likely to have been representative of OEF and OIF veterans with AUD. Further, as Tofighi et al [51] point out in their recent review, access barriers to technology (eg, smartphone ownership, connectivity) often mirror the very barriers to treatment that apps are attempting to overcome. Future studies must consider how best to disseminate treatment apps to those who need them and how to overcome technology and connectivity barriers. Our sample was small and composed of non–treatment-seeking OEF and OIF veterans who were aged 18 to 55 years and enrolled in VA care; our findings may not generalize to other OEF and OIF veterans, veterans of other service eras, or individuals in the community.

This pilot study represents one of the first studies to evaluate the effects of an interactive mHealth app for alcohol misuse in a community sample of veterans. Participants’ use of the app and alcohol-related outcomes were followed for 6 months, longer than many studies of apps addressing alcohol use. Acceptability and usability results provided essential and timely information to guide further refinement of Step Away (eg, modifications of text-heavy screens) and implementation of mHealth apps among veterans with alcohol misuse. If results are verified in a larger controlled trial, Step Away has the potential to improve access to alcohol-related care for a subset of the veteran population that is known to be reluctant to seek such care.

## Abbreviations

A-CHESS: Addiction Comprehensive Health Enhancement Support System
AUD: alcohol use disorder
DSM-5: Diagnostic and Statistical Manual of Mental Disorders, Fifth Revision
HDD: heavy drinking days
LBMI-A: Location-Based Monitoring and Intervention System for Alcohol Use Disorders
MET: motivational enhancement therapy
mHealth: mobile health
MINI: Mini International Neuropsychiatric Interview
OEF: Operation Enduring Freedom
OIF: Operation Iraqi Freedom
SIP-R: Short Inventory of Problems–Revised
SUS: System Usability Scale
TLFB: Timeline Followback
VA: Veterans Affairs
WHOQOL-BREF: abbreviated World Health Organization Quality of Life
